# Application of Platelet-Rich Fibrin and Concentrated Growth Factors as Carriers for Antifungal Drugs—In Vitro Study

**DOI:** 10.3390/jcm14145111

**Published:** 2025-07-18

**Authors:** Wojciech Niemczyk, Małgorzata Kępa, Jacek Żurek, Ali Aboud, Dariusz Skaba, Rafał Wiench

**Affiliations:** 1 Department of Periodontal Diseases and Oral Mucosa Diseases, Faculty of Medical Sciences in Zabrze, Medical University of Silesia, Pl. Traugutta 2, 41-800 Zabrze, Poland; dskaba@sum.edu.pl (D.S.); rwiench@sum.edu.pl (R.W.); 2 Medical Center of Innovation, Wroclaw Medical University, Krakowska 26, 50-425 Wroclaw, Poland; 3 Department of Microbiology, Faculty of Pharmaceutical Sciences in Sosnowiec, Medical University of Silesia in Katowice, Jagiellońska 4, 41-200 Sosnowiec, Poland; mkepa@sum.edu.pl; 4 Specialist Medical Practice—Clinical Periodontology, Żuradzka 15 Street, 32-300 Olkusz, Poland

**Keywords:** drug carriers, growth factors, in vitro, platelet-rich fibrin, candida, fungi

## Abstract

**Background**: Fungal infections, particularly those caused by *Candida* species, pose a serious threat to immunocompromised individuals, and therapeutic options are limited due to toxicity and resistance concerns. This in vitro study aimed to explore the feasibility of using liquid fractions of autologous platelet concentrates (APCs), namely concentrated platelet-rich fibrin (c-PRF) and liquid-phase concentrated growth factor (LPCGF), as carriers for antifungal drugs. **Methods**: The research was conducted in two phases: first, to evaluate the inherent antifungal properties of different APCs; and second, to assess their effectiveness as drug carriers for fluconazole and voriconazole against *Candida albicans*, *Candida glabrata*, and *Candida krusei*. **Results**: Results showed that APCs alone exhibited no direct antifungal effects. However, when combined with antifungal agents, notable inhibition zones were observed—especially with voriconazole against *C. krusei* and fluconazole against *C. glabrata* using c-PRF. Both c-PRF and LPCGF were compatible with the drugs and did not hinder clot formation. **Conclusions**: These findings suggest that APCs can act as effective vehicles for localized antifungal drug delivery and warrant further investigation for clinical application in treating fungal-related oral diseases.

## 1. Introduction

The preparation of platelet-rich fibrin (PRF) is a process that involves the coagulation of platelets in blood, resulting in the formation of a self-clotted preparation of blood-derived biomaterials. This objective is accomplished by means of contact activation of intrinsic coagulation pathways through centrifugation, with no additional coagulation factors being introduced [1]. Consequently, the preparation protocol has undergone substantial streamlining, resulting in a clot that can be managed with ease using forceps. PRF is further modified into two types: A-PRF, an advanced type, is expected to contain greater numbers of white blood cells and concentrated growth factors (CGF). It is prepared under a facilitated intrinsic coagulation cascade [2]. In recent years, significant advancements have been made in the field of PRF formulation, with a particular focus on the development of injectable PRF (i-PRF) [3]. CGFs are available in diverse forms and have been classified into two categories based on their state of existence: gel-phase concentrated growth factor (GPCGF) and liquid-phase concentrated growth factor (LPCGF). At present, GPCGF are more frequently utilized in the domain of hard tissue regeneration. Conversely, LPCGFs find application in wound healing, periodontal tissue regeneration, and the treatment of temporomandibular joint disorders owing to their exceptional mobility [4]. The liquid fractions of autologous platelet concentrates are distinguished by elevated concentrations of growth factors and leukocytes. Consequently, these fractions demonstrate a heightened microbial capacity in comparison to solid APCs [5,6].

In recent years, studies have shown the potential of using APCs as drug carriers. APCs provide a three-dimensional fibrin network that can act as a reservoir for drug molecules, allowing for sustained drug release as the matrix gradually degrades. Since there are many indications for APCs, some of which may have additional fungal infections, it seems rational to use APCs as carriers for antifungal drugs [7]. Nevertheless, to date, there have been no studies testing the possibility of using antifungal drugs for this purpose.

Fungal infections have become a significant global health problem, emerging as a primary cause of morbidity and mortality in immunocompromised patients. In the past 4 years, the Leading International Fungal Education (LIFE) portal has determined the global burden of severe fungal infections in over 80% of the current world population [8]. The number of currently available antifungal drugs is limited. A significant proportion of these organisms have demonstrated resistance to the available treatments. An important solution in the context of increasing drug resistance is to use drugs topically rather than systemically [9].

The existing antifungal medications can be categorized into three primary classes. These classes are polyenes, azoles, and echinocandins [10]. During the 1990s, there was a notable expansion of the antifungal armamentarium, with the introduction of two novel azole agents: fluconazole and itraconazole. These agents prompted a shift in the approach to treating numerous fungal infections. However, resistance to fluconazole soon emerged in immunosuppressed patients who received prolonged treatment. Consequently, the development of second-generation triazole agents has been a major focus over the past decade. The first of these new agents was voriconazole, a synthetic derivative of fluconazole. The modification of the triazole ring led to an augmentation in activity when compared with that of fluconazole [11].

The objectives of this in vitro study were as follows:

1. To test whether the addition of antifungal drugs to APCs would result in prolonged drug release, which could indicate drug incorporation into the fibrin network.
2. To assess whether autologous platelet concentrates allow for improved antifungal properties of fluconazole or voriconazole.
3. Whether there is a significant difference between the use of PRF and CGF in terms of the duration of drug release and the incorporation of antifungal activity.

## 2. Materials and Methods

**Figure 1:**
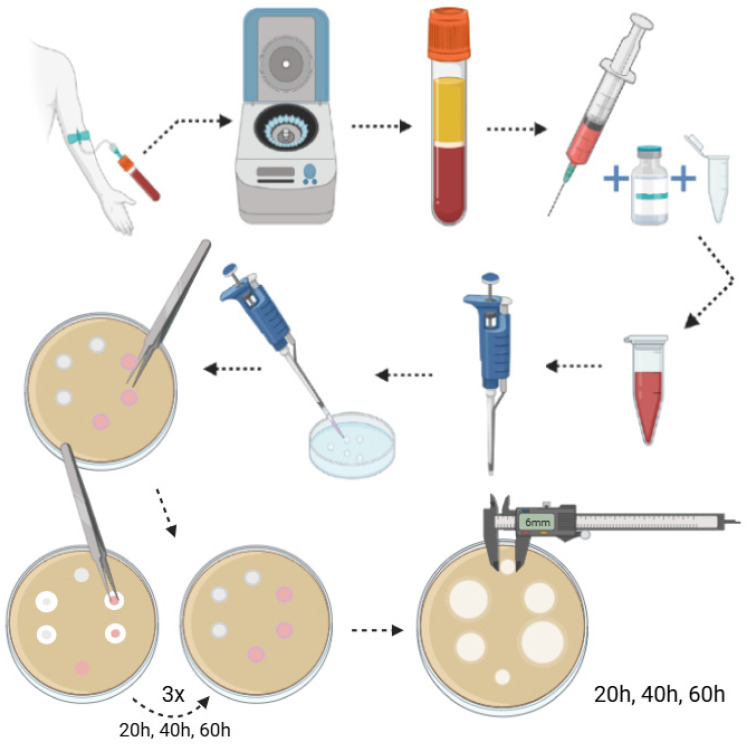
Diagram illustrating the basis of the methodology for phase two.

The study was meticulously divided into two phases. In the initial phase, a comparative analysis was conducted on the antifungal properties of various fractions of autologous platelet concentrates. The subsequent phase was designed to assess the feasibility of utilizing APCs as antifungal medications. The methodology of phase two is presented in the figure below (Figure 1).

### 2.1. Population and Study Design

The Bioethics Committee of the Silesian Medical University in Katowice granted approval for the study on 18 February 2025. The approval was documented under the reference number BNW/NWN/0052/KB1/10/25. The research was conducted in accordance with the European Committee on Antimicrobial Susceptibility Testing (EUCAST) standards, employing the Kirby–Bauer disk-diffusion method. In accordance with the inclusion and exclusion criteria delineated in Table 1, a total of 10 team members and volunteers provided blood samples for the study.

### 2.2. Phase One—Antifungal Properties of Specific APCs

#### 2.2.1. Phase One Groups

Antifungal properties were tested on Sabouraud agar with dextrose on a reference strain of *C. albicans* (ATCC 10231). Six groups were formed to test the antifungal properties:

1. control group—sterile disk with saline;
2. sterile disk with c-PRF (concentrated PRF);
3. sterile disk with LPCGF;
4. sterile disk with PRP (platelet-rich plasma);
5. sterile disk with i-PRF;
6. sterile disk with PPP (platelet-poor plasma).

#### 2.2.2. Phase One APC Preparation

Blood samples were collected using butterfly needles, gauge 21G (0.8 mm diameter) and 19 mm in length (Biomedico, Piaseczno, Poland). For the preparation of c-PRF, i-PRF, and LPCGF, 9 mL CDRICH plastic tubes (16 × 100 mm) without any additives were utilized. A water test was performed to confirm the absence of additional chemicals in the tube. For the preparation of PRP and PPP, 9 mL glass tubes with sodium citrate were used. For c-PRF, i-PRF, PRP, PPP the iFuge C4000 NXT centrifuge (Silfradent Srl, Forlì, Italy) was used.

Centrifuge settings:

− c-PRF were 8 min, RCF-max 2000 g [12];
− i-PRF were 3 min, RCF-max—60 g [3];
− PRP and PPP were at first 10 min, RCF-max 160, and afterwards 15 min, RCF 250 g [13].

For LPCGF, the Medifuge MF200 centrifuge (Silfradent Srl, Forlì, Italy) was used. The settings to obtain LPCGF consisted of a sequence of four protocols: acceleration × 30′, 2700 rpm × 2′, 2400 rpm × 4′, 2700 rpm × 4′, 3000 rpm × 3′, de-acceleration × 36′, centrifuge stopping [14].

### 2.3. APC Preparation for Second Phase

#### 2.3.1. Materials and Centrifugation Settings

In phase two, both LPCGF and c-PRF were achieved through the utilization of the same methodologies and devices employed in phase one.

#### 2.3.2. Drug Incorporation into APCs

Both c-PRF and LPCGF were removed from the tubes using a syringe and needle, which was positioned in the buffy coat zone at the border with the red blood cell layer. A volume of 0.5 milliliters of platelet concentrate was extracted from each tube. Solutions of fluconazole and voriconazole were added to APCs obtained by centrifugation. The exact amounts of antifungal agents were measured using a sterile automatic pipette. The preparations were subsequently mixed in a 2-mL Eppendorf tube. Subsequent to the mixing process, 20 µL of each preparation was collected and applied to a sterile disk (BLANK DISCS, Oxoid Ltd. Basingstoke, UK) with a diameter of 6 mm.

### 2.4. Fungal Strains

The table below shows the planned fungal strains to be used in the study together with the medium used (Table 2).

#### ATCC—American Type Culture Collection

The strains were obtained from the American Type Culture Collection (ATCC, Manassas, VA, USA) and are part of the strain bank of the Department of Microbiology, Faculty of Pharmaceutical Sciences in Sosnowiec, Medical University of Silesia in Katowice, Poland. The strains are stored at a temperature of −80 °C in a solution of tryptic-soy broth and glycerol.

### 2.5. Microbiological Analysis

Each microbial strain was individually cultured in a 90 mm diameter Petri dish containing 4 mm-thick agar medium (approximately 25 mL) sourced from bioMérieux (Marcy-l’Étoile, France). The agar surface was maintained dry and uniformly smooth. Following 24 h of aerobic incubation at 37 °C, a portion of the colony was collected from the agar surface and suspended in a sterile 0.9% sodium chloride (NaCl) solution. The number of viable cells in the suspension used was counted using a Densi-La-Meter II densitometer (ERBA LACHEMA, Prague, Czech Republic) at 525 nm. A McFarland optical density of 0.5 was used, corresponding to approximately 1–5 × 10^6^ CFU/mL.

Fifteen minutes after preparing the suspension, a sterile cotton swab was dipped into it. Using this swab, each strain was individually spread in three directions across the surface of Sabouraud dextrose agar (bioMérieux, Marcy-l’Étoile, France) to ensure even distribution. Antifungal compound (APC) disks were placed on the agar within 15 min following inoculation.

After each photographic session—conducted at 20-h intervals—the disks were relocated onto freshly inoculated Petri dishes, and the same procedure was repeated. This cycle continued until no inhibition zones larger than 6 mm were visible across the plate.

All cultures were incubated under aerobic conditions at 35 ± 2 °C in a MIR-262 SANYO incubator (Etten-Leur, The Netherlands). The antimicrobial effect was evaluated by measuring the diameter of the inhibition zones.

### 2.6. Study Groups

The study included eight experimental groups, as outlined in Table 3. Each group was tested in triplicate using separate Petri dishes. Within each dish, all groups corresponding to a specific APC and fungal strain were represented. To minimize variability due to individual differences, each dish used APCs derived from the same donor. However, for each replicate of the experimental setup, blood products from different participants were utilized.

**Figure 2:**
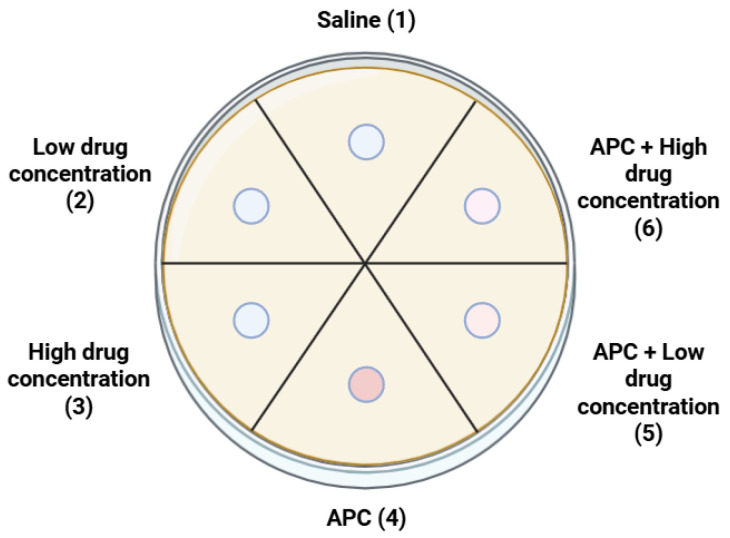
Arrangement of groups on the dishes.

The sequence of the dishes remained constant. The following figure illustrates the distribution of groups on the dish (Figure 2).

### 2.7. Measurement of Inhibition Zones

At 20, 40, and 60 h of incubation, the Petri dishes were taken out of the incubator and photographed using a Panasonic DMC-G80 Lumix camera (Osaka, Japan) fitted with a Lumix Panasonic H-FS 2060 12–60 mm micro-HD lens, positioned 30 cm above the plate at a perpendicular (90-degree) angle. The camera was mounted on a tripod to ensure consistent framing. All images were captured under identical conditions.

Once all photographs had been taken, the diameters of the inhibition zones surrounding each disk were measured. Image analysis was performed using the ImageJ—Fiji software (version 1.53j, US National Institutes of Health, Bethesda, MD, USA), with the final measurements recorded on 10 April 2025. The results were expressed in millimeters (mm).

The measurement was only taken after calibrating the size from left to right of the boundary of the microbial inhibition zone parallel to the base of the image so that the line passes through the center of the diffusion disk. The results thus obtained were recorded and then statistically evaluated.

### 2.8. Statistical Analysis

The results were presented as mean and standard deviation (M ± SD). Statistical differences were assessed using the analysis of variance (ANOVA) and the Tukey post hoc test. Furthermore, a Student’s *t*-test was employed to compare the two variables. A *p*-value ≤ 0.05 was considered to indicate a statistically significant difference. The statistical analysis was performed using Statistica, v. 7.1 PL (StatSoft Poland, Krakow, Poland).

## 3. Results

### 3.1. Antifungal Properties of APC Without Drug Addition

**Figure 3:**
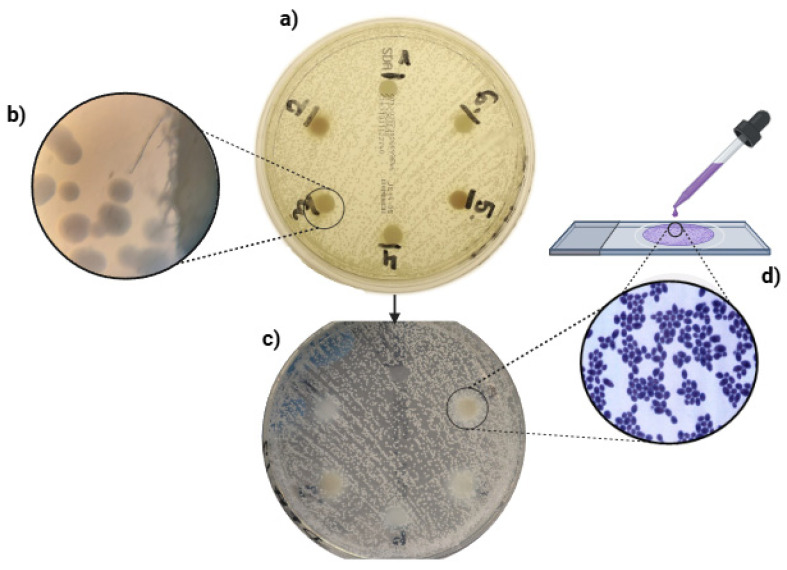
(**a**) Photograph of the dish 20 h after incubation. (**b**) Photograph of microscope view at 200× magnification. (**c**) Photograph of the dish after removal of the disks showing the opacity of the substrate. (**d**) Photograph of microscope view at 1000× magnification after Gram staining.

In the initial phase of the study, the antifungal properties of the various fractions of autologous platelet concentrates were examined (c-PRF, LPCGF, PRP, i-PRF, PPP). However, no inhibition zones were observed in any of the groups under investigation. However, a convex agar turbidity manifested in the vicinity of groups 2, 3, 4, 5, and 6. Subsequent microscopic examination of the disks revealed the presence of Gram-positive microorganisms, which were identified through the application of Gram’s staining technique. At both 200- and 1000-fold approximations, yeast cells of *C. albicans* (ATCC 10231) were the only visible elements under microscopic examination. These results were consistent with those obtained through Gram staining. The results are illustrated below (Figure 3).

### 3.2. Clot Formation After Addition of Drugs

Following the amalgamation of precise quantities of pharmaceutical agents with autologous platelet concentrates, the stability of the mixture was assessed. Specifically, the study examined whether the mixture would persist in a liquid state or if a clot would form. Subsequent to a 15-min period, the consistency of the substance was examined in a 2 mL Eppendorf tube. For mixtures where lower antifungal drug concentrations were used, 0.55 mL of solution were used, while for mixtures where higher antifungal drug concentrations were used, 0.625 mL of solution were used. The study’s findings revealed that none of the concentrations of the drugs tested exhibited interference with clot formation. The Eppendorf tubes were set on a shaker (VORTEX Genius 3 Shaker, Barcelona, Spain) to maintain the contents in a liquid state for an extended period, thereby facilitating the efficient execution of the study.

### 3.3. Antifungal Effect of APCs as Carriers for Drugs

#### 3.3.1. Fluconazole

**Figure 4:**
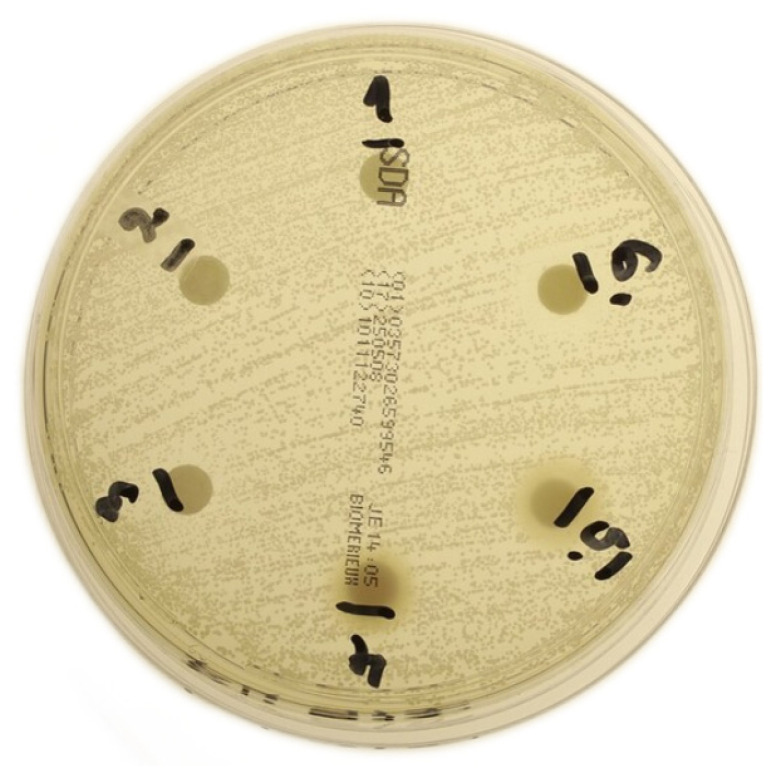
Photo showing visible zones of inhibition with fluconazole disks on c-PRF carrier on *C. glabrata* strain.

In the experimental groups administered with fluconazole, a zone of inhibition was observed in only one group. This was a group where the drug carrier was c-PRF in a Petri dish with a *C. glabrata* strain. This outcome was consistent across all three replicates. The zones of inhibition were observed exclusively surrounding the disks in groups 5 and 6, which utilized c-PRF in conjunction with low- and high-concentration drugs, respectively (Figure 4). The zone of inhibition for group 5 was 7.04 mm (±0.21 mm), and for group 6 it was 10.16 mm (±0.32 mm). Subsequent to the transfer of the disks to a novel medium and a subsequent reading of the results after 40 h, no zones of inhibition were observed. These findings suggest that the antifungal effect of fluconazole when delivered via c-PRF is weak and of short duration.

**Figure 5:**
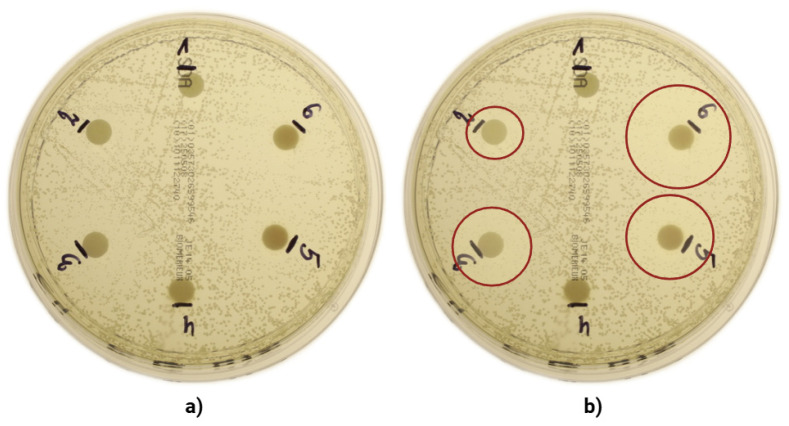
(**a**) Photograph showing the LPCGF carrier on a *C. albicans* strain after 20 h. (**b**) Highlighted zones of fungal colony dilution.

In the other groups, a decrease in fungal colony density was observed in the surrounding area of the disks with *C. albicans* and *C. glabrata* strains. However, the presence of resistant strains of *Candida* on the surfaces of these disks precludes the interpretation of these zones as zones of inhibition. However, Figure 5 demonstrates that, in general, there was a greater visible zone of dilution of fungal colony densities around the media disks.

#### 3.3.2. Voriconazole

**Figure 6:**
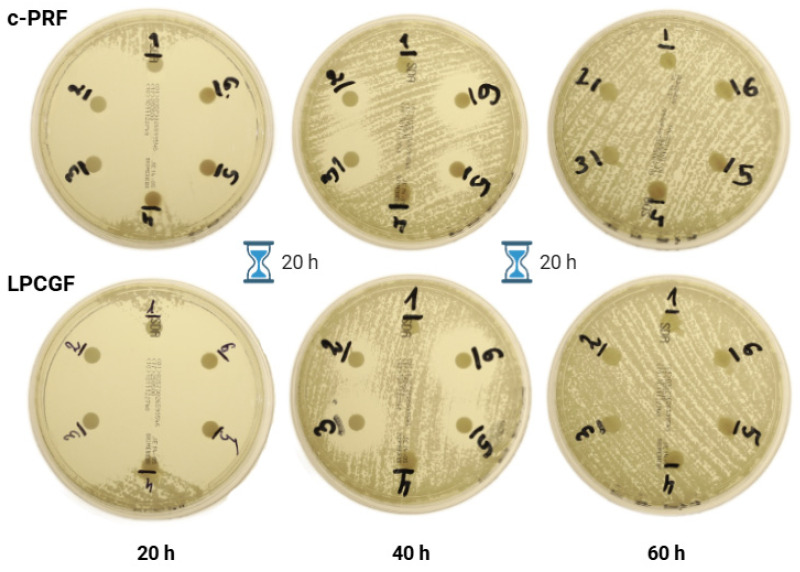
Photographs showing time-dependent inhibition zones for both carriers.

The application of voriconazole resulted in the appearance of zones of inhibition only among the *Candida krusei* strain (Figure 6).

The results for each group, presented as mean with standard deviation, are shown in Table 4. Statistical analysis showed that carrier application significantly (*p* < 0.05) increased the zones of inhibition around the disks with additional carrier application (both c-PRF and LPCGF).

Subsequent Student’s *t*-test analysis, comparing the efficacy of the various carriers, yielded variable outcomes. Specifically, at 20 h with a low concentration of incorporated drugs, and at 40 h with a high concentration of drugs, no statistically significant differences were observed between the groups. However, a comparison of the other groups revealed significant differences. When evaluated after 20 h, LPCGF demonstrated significantly superior outcomes when employed as a vehicle for elevated drug concentrations. Conversely, after 40 h, the c-PRF carrier exhibited a significantly larger zone of inhibition at lower drug concentrations. The results of the Student’s *t*-test are shown in Table 5.

## 4. Discussion

### 4.1. General Results

In the initial phase of the study, none of the tested autologous platelet concentrates—including c-PRF, i-PRF, PRP, PPP, and LPCGF—demonstrated any inherent antifungal activity when applied independently. These findings are also confirmed by other studies. A systematic review by Moraschini et al. demonstrated that APCs possess a greater antibacterial than antifungal capacity [5]. Furthermore, an increase in proliferating *Candida* strains was evident around the APC disks.

In the subsequent phase, the incorporation of antifungal agents into the APCs resulted in the manifestation of quantifiable antifungal effects. The combination of fluconazole and c-PRF resulted in observable inhibition zones against *Candida glabrata*, with measurements of 7.04 mm at the lower drug concentration and 10.16 mm at the higher concentration. While the combination of fluconazole with c-PRF yielded observable inhibition zones against *Candida glabrata*, these effects were characterized by limited magnitude and duration. The observed zones of inhibition were found to be relatively diminutive in size, and the antifungal activity waned completely after a duration of 40 h. In addition, fluconazole demonstrated no quantifiable activity against *Candida albicans* or *Candida krusei* in any carrier medium. These results suggest that, while there may be some initial antifungal release, the efficacy is likely insufficient for meaningful clinical application in its current form. The restricted dissemination and the absence of persistent activity should be interpreted as minimal antifungal efficacy, rather than as a broadly significant outcome.

Voriconazole, however, demonstrated more pronounced results. When used in conjunction with either c-PRF or LPCGF, voriconazole demonstrated significant antifungal activity against *Candida krusei*. At the 20-h mark, inhibition zones exceeding 40 mm were observed for both carriers, though these zones diminished by 60 h, at which point no inhibition zones remained. Statistical analysis revealed that LPCGF exhibited heightened efficacy at elevated drug concentrations after 20 h, while c-PRF demonstrated superior outcomes at reduced concentrations after 40 h. Notably, the incorporation of antifungal agents into the APCs did not impede clot formation, thereby suggesting the physical and chemical compatibility of the carriers with the drugs.

The study concluded that while APCs lack inherent antifungal properties, they function effectively as vehicles for antifungal drug delivery, particularly in the case of voriconazole against *C. krusei*. Nevertheless, the findings of this particular study alone cannot be considered sufficient to guide the selection of the most appropriate pharmaceutical agent for a given fungal strain. The most crucial step is to perform an antimycogram, which serves as a fundamental basis for selecting the most suitable pharmaceutical agent.

The most frequently utilized antifungal agents are classified within the azole class. Over the course of several decades, these compounds have demonstrated a wide range of applications in the treatment of fungal infections and diseases. There has been an increasing interest in and use of these agents in clinical settings [15]. However, it is imperative to acknowledge the potential adverse consequences associated with the prolonged administration of azoles [16]. Topical application may reduce the undesirable effects.

It is imperative to note that the concentrations of antifungal medications utilized in this study were selected with meticulous care, guided by preceding research on their cytotoxic potential. This approach was implemented to ensure the biocompatibility of the medications with human tissues. It is noteworthy that fluconazole, at concentrations ten times higher than those utilized in this experiment, has been demonstrated to exhibit no cytotoxic effect on mouse embryonic fibroblast 3T9 cells or human embryonic kidney 293 cells [17]. This observation lends further credence to the safety profile of fluconazole in localized applications, particularly when utilizing APCs as delivery vehicles. In contrast, voriconazole has been reported to suppress osteoblast and fibroblast growth at concentrations above 100 μg/mL [18]. However, the current study employed significantly lower concentrations, thereby minimizing potential cytotoxic effects.

This balance between therapeutic relevance and biocompatibility reflects the translational intention of this investigation, while concurrently establishing the foundation for subsequent inquiries into optimized dosage regimens and release kinetics. Recent studies on the kinetics of drug release have demonstrated an initial burst release of approximately 80% of the incorporated drug within the first hour, followed by a gradual release over time. The sustained release phase has been demonstrated to guarantee protracted antimicrobial activity, thereby mitigating the emergence of fungal resistance [7,19].

In addition, voriconazole has been shown to induce osteoblast proliferation and enhance osteogenic activity in vitro. This paradoxical effect, where lower concentrations may stimulate bone cell activity while higher concentrations inhibit it, could be particularly relevant in the field of regenerative dentistry. Furthermore, studies such as those by Allen et al. suggest that voriconazole-induced periostitis may occur through fluoride-independent pathways, involving enhanced expression of cytokines that promote osteoblastic activity. These findings are consistent with the regenerative potential of platelet-based products and support the hypothesis that voriconazole-loaded APCs could not only treat fungal infections but may also positively influence bone healing and regeneration [20].

As demonstrated by Straub et al., the selection of centrifugation protocol exerts a substantial influence on the characteristics of APC as a carrier for antibiotics. A series of experiments were conducted in order to assess the efficacy of three distinct protocols. Protocol A involved 1300 rotations per minute (RPM) at an amplitude of 8 min, with an RCF-max setting of 208 g. Protocol B involved 2300 RPM at an amplitude of 12 min, with an RCF-max setting of 652 g. Protocol C involved 1500 RPM with an amplitude of 14 min, and an RCF-max setting of 276 g. The findings of the experiments demonstrated that the protocol designated as B resulted in the most significant inhibition zone [21]. This study implemented a protocol to obtain c-PRF, which closely mirrors protocol B from the aforementioned study, given the utilization of high RCF values.

A considerable number of articles have previously explored the potential of utilizing autologous platelet concentrates as carriers for various pharmaceutical agents, including antibiotics [19,21,22,23,24,25,26,27,28,29,30,31,32,33,34,35,36,37,38,39,40,41,42,43,44], silver nanoparticles [45], and tranexamic acid [46]. To the authors’ knowledge, however, this is the first study to employ the combination of antifungal drugs and APCs. In the context of antibiotic administration, studies employing scanning electron microscopy (SEM) have demonstrated that antibiotic molecules become integrated into the fibrin network, thereby facilitating the controlled release of the antibiotic as it undergoes degradation within the body [19]. The observation of increased zones of inhibition at 40 h suggests the potential for a similar process to occur in the context of antifungal medications. The findings of this study suggest that the carriers demonstrate a certain degree of superiority. However, the authors of the study propose a hypothesis that suggests the absence of the disks may in fact enhance the efficiency of drug release. Notwithstanding, the utilization of the well-diffusion method was eschewed. This approach precludes the transference of the pertinent substance from the wells to the novel substrate following a 20-h period.

### 4.2. Limitations

It is imperative to acknowledge the limitations of this study when formulating conclusions and developing future applications. The most significant limitation pertains to the microbiological nature of the study, as no studies have been conducted to ascertain interactions between APCs and the drugs under investigation. While the incorporation of antibiotics into the fibrin network has been charted, this has not yet been studied for antifungal drugs. Another salient limitation pertains to the variability inherent in the blood parameters of the study volunteers. To mitigate the impact of individual variability, further research is necessary, either through additional studies or by expanding the study population. While the study makes a comparison between different APCs as carriers, it is recommended that future research focuses on comparing APCs to other substances as carriers. Another methodological limitation is the exclusive use of inhibition zone measurements to assess antifungal efficacy. While this approach is well-suited for evaluating the release and diffusion of antifungal agents from platelet concentrate-based carriers, it provides primarily qualitative and semi-quantitative data. The incorporation of colony-forming unit (CFU) enumeration, particularly in suspension-based models, would facilitate more precise quantification of viable fungal cells and provide complementary insights into fungistatic or fungicidal effects. It is recommended that subsequent studies integrate both diffusion-based and quantitative CFU methodologies to comprehensively assess antifungal activity. Furthermore, studies focusing on drug release kinetics are also lacking in determining the exact properties of APCs as carriers for antifungal drugs. The authors of this study utilized reference strains; however, the utilization of wild-type strains cultured from the microbiome of patients should be a worthwhile topic for future research. Furthermore, it is important to acknowledge that the results obtained under laboratory conditions may not fully reflect the true efficacy of PRF and CGF as drug carriers in clinical settings. The influence of the tissue microenvironment, interactions with host cells, and immunological processes may significantly alter the dynamics of the release of active substances.

### 4.3. Future Implications

#### 4.3.1. Periodontitis and Periimplantitis

Chen et al. in their analysis showed that specific fungal taxa play a role in periimplantitis [47]. Similar results were obtained by other authors in their studies [48,49]. In addition, it has been concluded that there is a strong association between the presence of *Candida* species and periodontal diseases [50]. A multitude of factors have been identified as contributing to the development of periodontitis in conjunction with *Candida* species. Among these factors are systemic diseases that lead to immunosuppression and changes in the oral environment, such as cigarette smoking. While a persistent, substantial surge in the identification rate of *Candida* species in patients diagnosed with periodontitis has not been universally corroborated, there exists substantiated documentation correlating the presence of *Candida* species to the severity of the disease, and its potential to exacerbate the condition. The mechanisms through which *Candida* species contributes to periodontitis are multifaceted, involving interactions across kingdoms with periodontal pathogens, alterations in the local or systemic milieu that favor the virulence of *Candida* species, and interactions between *Candida*-bacteria and host immunity [51]. In order to enhance the effectiveness of non-surgical periodontal treatment, the utilization of adjunctive treatments has been proposed. These adjunctive treatments include the use of antibiotics, antiseptics, or photodynamic therapy, with the objective of achieving enhanced decontamination of the root surfaces [52,53,54]. A systematic review by Niemczyk et al. demonstrated that the utilization of i-PRF as adjuvant therapy for periodontitis results in substantial improvements in clinical parameters [55]. In the future, it will be possible to trial the use of i-PRF incorporated with antifungal agents to assess the improvement in outcome.

#### 4.3.2. Lichen Planus

Oral lichen planus (OLP) is a chronic inflammatory disorder that affects the mucosa of the oral cavity. The disorder manifests in a variety of clinical presentations, including reticular, atrophic, erosive, or ulcerative lesions [56]. A meta-analysis by Gupta et al. demonstrated the efficacy of i-PRF in the treatment of OLP, showing comparable results to current steroid injections [57]. Similar results were obtained by Niemczyk et al. in a systematic review of the same issue [58]. The meta-analysis conducted by Rodriguez-Archilla and Fernandez-Torralbo revealed that more than one-third of OLP lesions are infected by *Candida* species, thereby modifying their biological behavior [59]. Parlatescu et al. postulate, on the basis of their retrospective study, that a routine screening of OLP patients for oral candidiasis and a preventive antifungal strategy in the OLP treatment schedule is necessary [60]. The combination of i-PRF and the antifungal agents it contains could be an ideal combination for cases of patients with fungal infection of the erosive form of lichen planus.

#### 4.3.3. Osteonecrosis of the Jaw

Osteonecrosis of the jaw (ONJ) is a rare condition that manifests as one or more necrotic bone lesions. These lesions are exposed or can be probed through an intraoral or extraoral fistula in the maxillofacial region. They persist for at least 8 weeks without response to appropriate therapy [61]. In recent years, it became increasingly evident that bone colonization with bacteria and possibly also fungi plays an important role in the pathogenesis of medication-related osteonecrosis of the jaw (MRONJ) [62]. It is important to recall that, besides bacteria, fungi, especially *Candida* spp., have also been found in bisphosphonate-related osteonecrosis of the jaw (BRONJ) lesions, which deserves attention when deciding on antimicrobial therapy [63,64,65,66,67]. If a fungal infection is found, it may be reasonable to use platelet-rich fibrin incorporated with antifungal drugs. Due to necrosis and poor blood supply to the necrotic area, systemic antifungal treatment may not be beneficial [7]. However, the need for antibiotic therapy should not be ignored; antifungal treatment should only be an adjuvant therapy to antibacterial therapy.

#### 4.3.4. Diabetic Foot Ulcers

*Candida* species, commensal residents of human skin, are recognized as the causative agents of cutaneous candidiasis across various body surfaces. Individuals with compromised immune systems, particularly those with immunosuppressive conditions, are at elevated risk of contracting this infection. Diabetes mellitus, a significant metabolic disorder, has emerged as a critical factor inducing immunosuppression, thereby facilitating *Candida* colonization and subsequent skin infections [68]. A considerable number of diabetic patients encounter complications due to angiopathy, neuropathy, and immune dysfunction, manifesting as diabetic foot ulcers (DFU) and diabetic foot infections (DFI). These complications can lead to lower limb amputation and, in certain cases, mortality. The utilization of antifungal medications as an ancillary component of treatment regimens has been demonstrated to markedly enhance the healing process of diabetic foot infections in patients with an underlying fungal infection [69]. This is of particular significance, as there is ample evidence supporting the occurrence of additional fungal infections in one out of every three patients diagnosed with diabetic foot infections. Furthermore, the analysis revealed that the predominant fungal strains were identified as *Candida* species [70,71]. The utilization of autologous platelet concentrates as a healing agent for DFUs has been a customary practice for an extended period. An analysis by Yang et al. demonstrated the superiority of healing with CGF over the use of PRF and PRP [72]. The potential application of APCs as carriers for antifungal drugs could facilitate enhanced healing in patients with fungal infections.

## 5. Conclusions

This study demonstrates that while autologous platelet concentrates do not exhibit direct antifungal activity, they can effectively serve as carriers for antifungal drugs such as fluconazole and voriconazole. Specifically, the combination of voriconazole with c-PRF or LPCGF demonstrated significant inhibition of *Candida krusei*, indicating the potential for targeted local therapy to be clinically relevant. Incorporation of fluconazole into c-PRF exhibited only a transient and localized effect against C. glabrata, underscoring the necessity for subsequent optimization of carrier concentration, drug loading, and delivery strategies prior to clinical translation. The integrity of both types of carriers was maintained without affecting clotting properties, supporting their viability for local delivery systems. These findings suggest potential avenues for future applications in the management of oral fungal infections, including periodontitis, lichen planus, and osteonecrosis of the jaw, particularly in settings where systemic antifungal treatment is limited or ineffective. However, further studies are necessary to validate these in vitro outcomes in clinical settings. These additional studies should particularly focus on drug release kinetics and patient-derived fungal strains.

## Figures and Tables

**Table 1:** Inclusion and exclusion criteria.

Inclusion Criteria:	Exclusion Criteria:
− Minimum age of 18 years − No systemic disease − Platelet count exceeding 200,000 per mm^3^ of blood (verified through lab tests conducted no more than 48 h before sample collection) − Provided informed consent for the withdrawal of 30 mL of blood	− Received systemic antifungal therapy in the past 6 months − Currently pregnant − Alcohol consumption within the last 7 days − Lactation − Vaccinated within the last 3 months − Menstruation − Known hypersensitivity to any substances used in the experiment − Taking NSAIDs within the last 8 weeks

**Table 2:** The reference strains used and the media for their culture.

Fungal Strain	Culture Media	ATCC Number
*Candida albicans*	Sabouraud Dextrose Agar	10231
*Candida glabrata*	Sabouraud Dextrose Agar	15126
*Candida krusei*	Sabouraud Dextrose Agar	14243

**Table 3:** Table showing the study groups with exact drug concentrations.

Drug Incorporated into APC	Amount of Drug Added per mL of APC (c-PRF/LPCGF)	Concentration of Drug in APC
Fluconazole (Fluconazole B. Braun)	0.1 mL (0.2 mg)	0.002 mg/mL
	0.25 mL (0.5 mg)	0.004 mg/mL
Voriconazole (Voriconazole Genoprim ^®^)	0.1 mL (1 mg)	0.009 mg/mL
	0.25 mL (2.5 mg)	0.02 mg/mL

APC—autologous platelet concentrations, c-PRF—concentrated platelet-rich fibrin, LPCGF—liquid phase concentrated growth factor.

**Table 4:** Results of inhibition zones on *C. krusei* strain presented as mean with standard deviation.

APC	Time of Measurement	Group 1	Group 2	Group 3	Group 4	Group 5	Group 6
c-PRF	20 h	-	41.2 mm (±1.03 mm)	44.55 mm (±1.14 mm)	-	43.81 mm (±1.13 mm)	45.89 mm (±0.67 mm)
	40 h	-	14.82 mm (±0.12 mm)	19.87 mm (±0.13 mm)	-	15.31 mm (±0.1 mm)	20.72 mm (±0.56 mm)
	60 h	-	-	-	-	-	-
LPCGF	20 h	-	41.08 mm (±1.33 mm)	46.59 mm (±1.06 mm)	-	42.5 mm (±1.51 mm)	47.22 mm (±0.79 mm)
	40 h	-	12.27 mm (±0.52 mm)	19.47 mm (±0.41 mm)	-	13.66 mm (±0.47 mm)	20.55 mm (±0.48 mm)
	60 h	-	-	-	-	-	-

APC—autologous platelet concentrations, c-PRF—concentrated platelet-rich fibrin, LPCGF—liquid phase concentrated growth factor, “-” marks values where the radius of the inhibition zone was 6 mm. In red are statistically significant values (*p* < 0.05) based on Welch’s *t*-test of corresponding groups with the same drug concentration.

**Table 5:** Results of the Student’s *t*-test for carrier comparison.

Variable Under Test	*p*-Value	Conclusion
Low drug concentration tested at 20 h	0.26	No significant difference
High drug concentration tested at 20 h	0.038	LPCGF superior
Low drug concentration tested at 40 h	0.019	c-PRF superior
High drug concentration tested at 40 h	0.42	No significant difference

c-PRF—concentrated platelet-rich fibrin, LPCGF—liquid phase concentrated growth factor.

## Data Availability

The data presented in this study are available on request from the corresponding author.
